# The Role of Connexin and Pannexin Channels in Perinatal Brain Injury and Inflammation

**DOI:** 10.3389/fphys.2019.00141

**Published:** 2019-02-27

**Authors:** Kelly Q. Zhou, Colin R. Green, Laura Bennet, Alistair J. Gunn, Joanne O. Davidson

**Affiliations:** ^1^Department of Physiology, The University of Auckland, Auckland, New Zealand; ^2^Department of Ophthalmology, The University of Auckland, Auckland, New Zealand

**Keywords:** connexin, pannexin, hemichannel, ischemia, inflammation, inflammasome, ATP

## Abstract

Perinatal brain injury remains a major cause of death and life-long disability. Perinatal brain injury is typically associated with hypoxia-ischemia and/or infection/inflammation. Both hypoxia-ischemia and infection trigger an inflammatory response in the brain. The inflammatory response can contribute to brain cell loss and chronic neuroinflammation leading to neurological impairments. It is now well-established that brain injury evolves over time, and shows a striking spread from injured to previously uninjured regions of the brain. There is increasing evidence that this spread is related to opening of connexin hemichannels and pannexin channels, both of which are large conductance membrane channels found in almost all cell types in the brain. Blocking connexin hemichannels within the first 3 h after hypoxia-ischemia has been shown to improve outcomes in term equivalent fetal sheep but it is important to also understand the downstream pathways linking membrane channel opening with the development of injury in order to identify new therapeutic targets. Open membrane channels release adenosine triphosphate (ATP), and other neuroactive molecules, into the extracellular space. ATP has an important physiological role, but has also been reported to act as a damage-associated molecular pattern (DAMP) signal mediated through specific purinergic receptors and so act as a primary signal 1 in the innate immune system inflammasome pathway. More crucially, extracellular ATP is a key inflammasome signal 2 activator, with purinergic receptor binding triggering the assembly of the multi-protein inflammasome complex. The inflammasome pathway and complex formation contribute to activation of inflammatory caspases, and the release of inflammatory cytokines, including interleukin (IL)-1β, tumor necrosis factor (TNF)-α, IL-18, and vascular endothelial growth factor (VEGF). We propose that the NOD-like receptor protein-3 (NLRP3) inflammasome, which has been linked to inflammatory responses in models of ischemic stroke and various inflammatory diseases, may be one mechanism by which connexin hemichannel opening especially mediates perinatal brain injury.

## Introduction

Perinatal brain injury is associated with death or significant long-term neurodevelopmental impairment, affecting 1.15 million infants in the world in 2010 (Lee et al., 2013). It affects both term and preterm infants (born <37 weeks of completed gestation), with the greatest incidence in the most preterm infants (Larroque et al., 2008). In term born infants, perinatal brain injury is most often linked to hypoxia-ischemia (HI) (Vannucci, 2000; Shankaran et al., 2005), where the incidence of moderate to severe hypoxic-ischemic encephalopathy (HIE) is ~1–3 in 1,000 live births (Edwards et al., 2010). By contrast, in preterm infants, brain injury arises from the complex interaction of HI, infection/inflammation and preterm birth itself (Galinsky et al., 2018b). Strikingly, ~50% of extremely preterm infants (born <26 weeks of gestation) develop moderate to severe disability (Marlow et al., 2005).

Currently, there is only one established neuroprotective treatment for term babies born with HIE, which is therapeutic hypothermia. Clinically, this treatment is only partially effective, with a number needed to treat of ~8, meaning of 8 treated infants with HIE, one additional infant will survive without moderate to severe disability (Gunn et al., 2017; Natarajan et al., 2018). However, there are currently no proven treatments to reduce brain damage for preterm infants with HIE or perinatal infection/inflammation. Therefore, there is significant interest in developing complementary or novel treatments for perinatal brain injury after HI or infection/inflammation. For term infants, it is important to develop interventions with additive benefit to therapeutic hypothermia, as it is now standard therapy. Novel treatments are needed for term and preterm infection/inflammation.

After HI, there is a striking evolution of injury which progresses over time from injured to previously uninjured regions of the brain (Azzopardi et al., 1989; Thornton et al., 1998). The mechanisms behind this spread are not well-understood. Increasing evidence supports the opening of cell membrane channels after HI as one of the mediators of this propagation of injury, particularly connexin hemichannels, which are the unopposed half of a gap junction, forming large conductance channels in the cell membrane (Davidson et al., 2012, 2014; Wang et al., 2014; Li et al., 2015). Pannexin channels may also open under pathological conditions, although they appear to be tightly regulated (Qiu and Dahl, 2009) and their role in the developing brain remains unclear. In this review, we examine the hypothesis that prolonged opening of connexin hemichannels leads to a cascade of injurious mechanism, potentially through inflammation by activation, amplification and perpetuation of the inflammasome pathway.

An inflammatory response is seen in perinatal brain injury after HI as well as infection/inflammation, and if chronic inflammation is established can have deleterious effects (Hagberg et al., 2015; Bennet et al., 2018). Hemichannels release adenosine triphosphate (ATP) (Kang et al., 2008; Chekeni et al., 2010; Orellana et al., 2011; Bennett et al., 2012), which may act as a damage associated molecular pattern (DAMP), but is also a key inflammasome activator signal. The release of ATP through connexin hemichannels can perpetuate inflammation through purinergic signaling (Pedata et al., 2016). Purinergic signaling has been associated with the activation of microglia (Li et al., 2011; Kaiser et al., 2016) and the inflammasome complex—a multi-protein complex involved in initiating an innate immune system inflammatory response (Feng et al., 2015). This review will dissect the evidence for these pathways downstream of connexin hemichannel and pannexin channel opening and how they contribute to inflammation. One other membrane pore forming protein, Gasdermin D, has been implicated in the inflammasome pathway (Groslambert and Py, 2018; Kerur et al., 2018), but that channel is very small in comparison with the pannexin and connexin channels and appears to be primarily associated with apoptosis. As will become evident in this review, both *in vitro* and animal studies using connexin hemichannel or pannexin channel blockers, would suggest that other channels are likely to play minor roles in relation to ATP release, and inflammasome activation in particular.

## Evolution of Injury

Perinatal brain injury after HI is an evolving process that can be characterized into four phases (Bennet et al., 2010; Davidson et al., 2015b). The primary phase of injury occurs during the HI insult itself, when the failure of oxidative metabolism results in anoxic depolarization, edema and necrosis (Wassink et al., 2018). After restoration of blood flow and oxygen supply, there is a period of apparent transient recovery when oxidative metabolism is at least partially restored, known as the latent phase (Davidson et al., 2018a). However, the latent phase is the key time when the deleterious mechanisms leading to the spread of brain injury may be initiated; for example, the opening of connexin hemichannels, which will be discussed further below (Davidson et al., 2012).

Following the latent phase, there is a delayed (“secondary”) deterioration of oxidative metabolism starting ~6–15 h after the insult (Azzopardi et al., 1989; Williams et al., 1991; Gunn et al., 1997). This phase is characterized by delayed cerebral energy failure followed by seizures and secondary cell swelling (Bennet et al., 2006; Davidson et al., 2015c). There is marked neuronal injury after HI at term, with the majority of neuronal loss occurring during the secondary phase, through a continuum of necrosis-apoptosis and autophagy (Northington et al., 2007, 2011).

The tertiary phase is a period of repair and reorganization, persisting for weeks to years after the initial insult (Fleiss and Gressens, 2012). During this period, surviving cells in the brain can rewire, but there may be a low level of ongoing cell death due to the loss of trophic support and problems with connectivity (Ness et al., 2001; Romanko et al., 2004). Long-term impairment in perinatal brain injury may also be associated with epigenetic changes (Fleiss and Gressens, 2012), but also persistent inflammation (Bennet et al., 2018) as reported for other types of brain injury and degenerative diseases (Patterson and Holahan, 2012; Freeman and Ting, 2016).

## Inflammation in Perinatal Brain Injury

Perinatal brain injury associated with HI or infection/inflammation can trigger an inflammatory response. The innate immune response is the body's first line of defense against pathogens, reacting rapidly following exposure to invading organisms (Medzhitov, 2007). As part of the innate immune response, pattern recognition receptors (PRRs) expressed on immune cells recognize both the conserved molecular structures found on the pathogen known as pathogen-associated molecular patterns (PAMPs), and the endogenous signals released by damaged tissues known as danger-associated molecular patterns (DAMPs) (Medzhitov, 2007; Takeuchi and Akira, 2010).

The activation of PRRs initiates the inflammatory response leading to release of inflammatory cytokines, such as interleukin (IL)-1β (Turner et al., 2014). This early phase of inflammation targets the invading pathogens and/or clears injured tissue, which is beneficial to the host. However, this inflammatory response also leads to the death of uninjured neural cells in a process known as “bystander cell loss” (Hagberg et al., 2015). It is believed that the initial pro-inflammatory response is followed by anti-inflammatory and reparative processes, and either eventual resolution of inflammation or chronic inflammation (Gilroy and De Maeyer, 2015; Hagberg et al., 2015).

Elevated levels of inflammatory markers after birth are associated with adverse neurodevelopmental outcomes. A prospective cohort study of 73 term infants exposed to perinatal asphyxia showed that those who died or were diagnosed with cerebral palsy at a 1 year follow up, were associated with higher levels of IL-1, IL-6 and tumor necrosis factor (TNF)-α in heel-stick blood samples collected on the first or second day of birth (Foster-Barber et al., 2001). In the same cohort, followed up at 30 months of age, those with higher serum cytokine levels of IL-1β, IL-6, and IL-8 at birth were associated with abnormal cognitive and motor outcomes (Bartha et al., 2004). The extremely low gestational age newborns (born before 28 weeks of gestation) (ELGANs) study is a large multi-center observational study. In this cohort, elevated blood concentrations of inflammatory proteins, measured in the first few weeks of life was associated with mental and motor impairments at 2 years old (O'Shea et al., 2012) and adverse cognitive outcome at 10 years of age (Kuban et al., 2017), as well as increased risk of behavioral problems, including autism (Korzeniewski et al., 2018). Cytokine levels were not measured at later time points to determine if inflammation was sustained. However, a study by Lin and colleagues has suggested that perinatal brain injury is associated with long-lasting alterations to the inflammatory response (Lin et al., 2010). Peripheral blood mononuclear cells from a small cohort of preterm born children with periventricular leukomalacia induced cerebral palsy, were shown to have higher mRNA levels of inflammatory molecules, both before and after lipopolysaccharide (LPS) stimulation (Lin et al., 2010). Non-resolving inflammation could be the result of a prolonged or excessive inflammatory response, which may lead to the disruption of pathways that normally induce inflammatory resolution (Nathan and Ding, 2010; Bennet et al., 2018).

## Connexin Hemichannels

**Figure 1:**
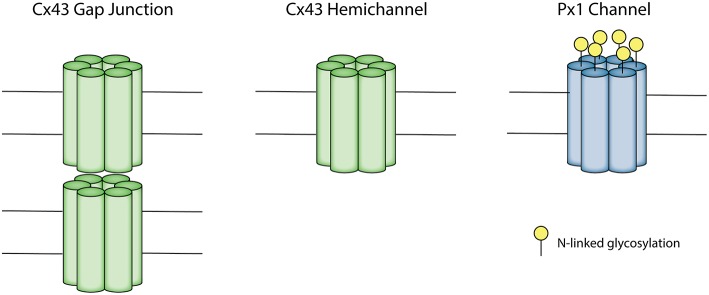
Schematic depicting the Connexin 43 (Cx43) gap junction and hemichannel and pannexin 1 (Px1) channel. A connexon consists of six connexin subunits. A Cx43 gap junction is made up of two opposing connexons, each contributed from two adjacent cells. A Cx43 hemichannel is an unopposed connexon. A Px1 channel is formed by six pannexin subunits. Px1 does not form gap junctions due to N-linked glycosylation on the extracellular loop.

Connexin gap junctions connect the intracellular space of two adjacent cells allowing for the exchange of ions and molecules (Kumar and Gilula, 1996; Alexander and Goldberg, 2003; Davidson et al., 2013a). A connexin hemichannel is comprised of six connexin subunits. Connexin hemichannels from adjacent cells dock together to form a gap junction (Unger et al., 1999; Davidson et al., 2013a; Leybaert et al., 2017) (Figure 1). Humans have 21 connexin genes, and 11 of these are expressed in the brain (Theis et al., 2005). Connexin 43 (Cx43) has been of particular interest in the brain as it is abundantly expressed, especially in astrocytes, microglia and microvascular endothelium (Dermietzel et al., 2000; Nagy et al., 2003; Davidson et al., 2013a). Under physiological conditions, prior to forming a gap junction, hemichannels at the cell surface have a low probability of opening, shown by low membrane permeability during resting conditions in cultured cells (Decrock et al., 2009). It is not known whether this is the case *in vivo*, as cells may be exposed to a wide range of stimuli influencing hemichannel opening (Sáez et al., 2005). When hemichannels do open though, they form a large non-selective membrane channel capable of passing molecules up to about 1 kDa in size (Evans et al., 2006). There is increasing evidence that dysregulated hemichannel opening can be detrimental and they are often referred to as “pathological pores” (Paul et al., 1991; Decrock et al., 2015; Willebrords et al., 2016).

Connexin hemichannels are involved in the spread of brain injury after HI or infection in the adult and immature brain (Davidson et al., 2012, 2013a) (studies are summarized in Table 1). They open in response to stimuli mimicking exposure to ischemia and inflammation *in vitro*. For example, in cultured cells, oxygen-glucose deprivation (OGD), metabolic inhibition, low extracellular Ca^2+^, and strong depolarization have all been proven to stimulate hemichannel opening (Contreras et al., 2002; Decrock et al., 2009; Orellana et al., 2010). Conditioned medium from LPS-activated microglia or TNF-α and IL-1β increased hemichannel activity in cultured astrocytes (Retamal et al., 2007). In addition, neuroinflammation induced by S. aureus intracerebral inoculation increased Cx43 hemichannel activity in astrocytes surrounding the abscess (Karpuk et al., 2011). The opening of connexin hemichannels allows for the influx of Na^+^, Cl^−^, and Ca^2+^ and the efflux of K^+^ ions. This increases cell permeability, leading to the depolarization of the cell, and cell lysis (Paul et al., 1991; Gómez-Hernández et al., 2003). Molecules, such as ATP, glutamate and aspartate can be released; all of these molecules are associated with injury (Ye et al., 2003; Gomes et al., 2005; Zhao et al., 2005; Kang et al., 2008). Additionally, Cx43 hemichannels are likely involved in the propagation of seizures following severe HI in near-term fetal sheep (Davidson et al., 2012).

**Table 1:** Summary of studies of Cx43 hemichannels and Px1 channels and purinergic receptors P2X4 and P2X7.

	**Species**	**Age**	**Paradigm**	**Hemichannel activity/expression**	**Drug/knockout**	**Drug/knockout effect**	**References**
**Connexin 43 (Cx43)**	Rat primary astrocyte culture	Embyronic day (E) 9	Metabolic inhibition	↑ Cx43 hemichannel opening during metabolic inhibition			Contreras et al., 2002
	Rat primary astrocyte culture	P1/2	Conditioned medium from LPS-activated microglia or TNF-α and IL-1β	↑ Cx43 hemichannel activity 24 h after treatment			Retamal et al., 2007
	Rat primary astrocyte culture	P1/2	Hypoxia and artificial cerebrospinal fluid medium mimicking ischemic conditions	↑ Cx43 hemichannel opening 1 h after reoxygenation			Orellana et al., 2010^*^
	Human microvascular endothelial cells		Hypoxic acidic ion-shifted ringer solution	↑ ATP release from Cx43 hemichannels during treatment and after reperfusion			Kim and Green, 2016^*^
	C6 glioma cells transfected with Cx43		Voltage activation of Cx43 hemichannels	↑ ATP release from Cx43 hemichannels			Kang et al., 2008
	Human adult retinal pigment epithelial cells		High glucose, TNF-α and IL-1β	↑ ATP release from Cx43 hemichannels	Peptide 5 (5–50 μM), administered at the same time as high glucose and cytokine treatment	↓ NLRP3 oligomerization ↓ Cytokine release	Mugisho et al., 2018
	Mouse acute brain slice	8–12 weeks	*S. aureus in vivo* intracerebral inoculation	↑ Cx43 hemichannel activity			Karpuk et al., 2011^*^
	Sheep	Near-term	Carotid artery occlusion	↑ mRNA expression (6 h after end of occlusion)	Peptide5 i.c.v. (50 μmol/kg over 1 h, then 50 μmol/kg over 24 h) started 90 min after end of occlusion	↑ Neuronal and oligodendrocyte survival Improved electroencephalogram recovery ↓ Seizure burden	Davidson et al., 2012; Galinsky et al., 2017
	Sheep	Near-term	Carotid artery occlusion		Peptide5 i.c.v. (50 μmol/kg over 1 h, then 50 μmol/kg over 24 h) started 3 h after end of occlusion	↓ Seizure burden	Davidson et al., 2015a
	Sheep	Near-term	Carotid artery occlusion		Peptide5 i.c.v. (50 μmol/kg/h) given 1 h before, and during occlusion only	No neuroprotection	Davidson et al., 2013b
	Sheep	Preterm	Umbilical cord occlusion		Peptide5 i.c.v. (50 μmol/kg over 1 h, then 50 μmol/kg over 24 h) started 90 min after end of occlusion	↑ Neuronal and oligodendrocyte Improved electroencephalogram recovery	Davidson et al., 2013a
	Rat	P7	Common carotid artery ligation and hypoxia	↑ Protein expression (24–48 h after HI)			Wang et al., 2014
	Rat	P7	Common carotid artery ligation and hypoxia	↑ Protein expression (8 h to 7 d after HI)	Gap 26 i.p. (50 μg/kg, 1 h) before HI	↓ Infarct size ↑ Motor and memory scores	Li et al., 2015
	Rat	P10	Acute hypoxia	No change (10 min to 35 d after HI)			Zeinieh et al., 2010
**Pannexin 1** **(Px1)**	Rat primary neurons	P20-25	N-methyl-D-aspartate receptor (NMDAR) activation	↑ Px1 channel opening			Thompson et al., 2008
	Rat primary neurons	P15-20	OGD	↑ Px1 channel opening during OGD			Thompson et al., 2006
	Mouse hippocampal slice	P13-14	High K^+^ medium	↑ Px1 channel opening			Santiago et al., 2011
	Mouse primary astrocytes		OGD		Probenecid (10 μM)	↓ IL-1β release ↓ NLRP3 protein expression	Jian et al., 2016
	Mouse acute brain slice	8–12 weeks	*S. aureus in vivo* intracerebral inoculation	↑ Px1 channel activity			Karpuk et al., 2011^*^
	Xenopus oocytes expressing Px1		High K^+^ medium	↑ ATP release during treatment from Px1 channels			Bao et al., 2004
	Rat primary astrocyte culture	P1/2	Hypoxia and artificial cerebrospinal fluid medium mimicking ischemic conditions	No Px1 channel opening at 1 h after reoxygenation			Orellana et al., 2010^*^
	Human microvascular endothelial cells		Hypoxic acidic ion-shifted ringer solution	↑ ATP release from Px1 channels during treatment but not after reperfusion			Kim and Green, 2016^*^
	Mice	Adult	MCAO		Mefloquine i.p. (1 mg/kg/d) given during the start of MCAO	↓ Infarction size ↑ Motor scores ^*^ No additive effect with P2X7R blockade	Cisneros-Mejorado et al., 2015a^*^
	Rat	Adult	MCAO		Probencid (i.v., 2 mg/kg) given before reperfusion	↑ Neuronal survival	Wei et al., 2015
	Mice	Adult	MCAO	↑ Px1 protein expression in females vs. male on ischemic and non-ischemic hemispheres	Probenicid i.p. (250 mg/kg) at 1.5 and 5 h	↓ Infarct volume (female only)	Freitas-Andrade et al., 2017
**Purinergic receptor (P2X4R)**	Rat hippocampal slice	P8-10	OGD	↑ Protein expression at 24 h			Cavaliere et al., 2003
	Mice	Adult	MCAO	↑ Protein expression at 6, 24, and 72 h	Global P2X4R knockout Myeloid cell P2X4R knockout	↓ Infarct volume ↓ IL-1β and TNF-α release ↓ Infarct volume (females only)	Verma et al., 2017
	Rat primary microglia	P2-3			TNP-ATP (20 μM)	↓ Change of microglia to rounded morphology ↓ IL-1β and TNF-α protein expression	Li et al., 2011
	Rat	P0	Hypoxia	↑ Protein expression from 4 h to 14 d			Li et al., 2011
	Rat	P3	Common carotid artery occlusion and hypoxia	↑ Protein expression 5 and 7 d after HI			Wixey et al., 2009
**Puringeric receptor (P2X7R)**	Neuronal culture		OGD		Brilliant Blue G (50 nM or 5 μM) during OGD	↓ Cell death	Arbeloa et al., 2012
	Rat	Adult	MCAO		Brilliant Blue G i.p. (30 mg/kg) during MCAO	↓ Neuronal loss ↓ Infarct volume	Arbeloa et al., 2012
	Rat	P0	Intrauterine asphyxia	↑ Protein expression immediately after asphyxia			Frizzo et al., 2010
	Rat	Adult	MCAO	↑ Protein expression 4 d after MCAO			Franke et al., 2004
	Rat	Adult	Common carotid and vertebral arteries occlusion	↑ Protein expression from 6 h to 7 d after insult	Brilliant Blue G treatment i.v. (50 mg/kg) administered daily, for 3 d immediately after occlusion	↓ Neuronal loss	Yu et al., 2013
	Rat	Adult	MCAO		A-438079 (3 μg, i.c.v.) or Brilliant Blue G (10 μg, i.c.v.), or Oxidized ATP (1 μg, i.c.v.), prior to HI	↓ Neuronal loss ↑ Motor performance	Chu et al., 2012
	Rat	Adult	MCAO	*De novo* expression of P2X7R on microglia			Melani et al., 2006
	Rat	Adult	Intracerebral hemorrhage		P2X7R siRNA i.c.v. (1,000 pmol)	↓ NLRP3 inflammasome activation ↓ IL-1β and IL-18 release	Feng et al., 2015
	Neuronal culture		OGD	↑ Protein expression after 3, 6, and 12 h of OGD			Ye et al., 2017
	Mice	Adult	Photothrombotic cerebral ischemia	↑ Protein expression from 1 to 5 d after insult	Brilliant Blue G (45.5 mg/kg, i.p.) given at 1, 3, and 5 d	↓ Infarct volume ↓ Protein expression of NLRP3, ASC, and caspase 1 p20	Ye et al., 2017
	Mice	Adult	MCAO		Brilliant Blue G (30 mg/kg twice per day, i.p.) during MCAO	↓ Infarction size ↑ Motor scores ^*^ No additive effect with Px1 blockade	Cisneros-Mejorado et al., 2015a^*^
	Mice	Adult	MCAO		P2X7R knockout	↓ Microglia activation	Kaiser et al., 2016

* *Studies that report interactions between connexin hemichannels, pannexin channels, and/or purinergic receptors*.

An upregulation of Cx43 has been shown to occur in adult and perinatal animals after HI and in adult human post-mortem studies of cerebral ischemia (Nakase et al., 2006, 2009; Davidson et al., 2012; Wang et al., 2014; Li et al., 2015). The timing of this Cx43 increase varies between studies, with Cx43 mRNA levels significantly increased at 6 h after carotid artery occlusion in near-term fetal sheep (Davidson et al., 2012). Protein levels progressively increased in the ischemic region from 8 h to 7 days after common carotid artery ligation and hypoxia (commonly referred to as Rice-Vannucci model of HI) in post-natal day (P)7 rats (equivalent to 33–34 weeks of human brain maturation) (Li et al., 2015). In the subventricular zone, however, Wang and colleagues reported Cx43 protein expression was only significantly increased from 24 h after the same insult in P7 rats (Wang et al., 2014). However, there was no change in hippocampal Cx43 protein expression at all time-points between 10 min and 35 days after acute hypoxia in P10 rats (equivalent to human term brain maturation) (Zeinieh et al., 2010). Thus, the changes in Cx43 expression may be dependent on species, brain region, type and/or severity of insult. It should be noted that, the Rice-Vannucci model of HI in rodents produces a unilateral infarction in the brain but only moderate systemic hypoxia (Rice et al., 1981), whereas bilateral carotid artery occlusion in near-term fetal sheep produces a global cerebral insult resulting in a watershed pattern of brain injury, without systemic hypoxia (Williams et al., 1992). The reader should note that protein assays and immunohistochemical labeling do not allow ready discrimination between gap junctions and hemichannels, or channel function.

The blockade of Cx43 hemichannels, however, has been neuroprotective in a variety of different animal models of perinatal HI (Davidson et al., 2012, 2014; Li et al., 2015). Intracerebroventricular (i.c.v.) infusion (50 μmol/kg over 1 h, then 50 μmol/kg over 24 h) of the Cx43 mimetic peptide—Peptide5, started at 90 min after the end of carotid artery occlusion in near-term fetal sheep, was associated with improved neuronal and oligodendrocyte survival and, electroencephalogram (EEG) recovery and reduced seizure burden (Davidson et al., 2012). Similarly, Peptide5 infusion following the same protocol as above in preterm fetal sheep after umbilical cord occlusion improved EEG recovery and reduced neuronal and oligodendrocyte loss (Davidson et al., 2014). Reduced infarct size and improved motor and memory scores were observed in Gap 26 treated P7 rats [a connexin mimetic peptide which blocks hemichannels and gap junctions (Wang et al., 2013)], given i.p., 50 μg/kg, 1 h before HI (Li et al., 2015).

Cx43 hemichannel opening *in vivo* has been shown to occur after ischemia (in the latent phase), but not during ischemia. Pre-administration of Peptide5 was not neuroprotective when given 1 h before and during ischemia, but was neuroprotective when given after ischemia (Davidson et al., 2012, 2013b). Supporting this, hemichannels in astrocyte cultures subjected to OGD have been shown to open 1 h after OGD (Orellana et al., 2010). In addition, carbenoxolone (100 μmol/L) inhibition of connexin hemichannels did not attenuate ATP release during OGD in hippocampal slices (Frenguelli et al., 2007). Both Cx43 hemichannel and pannexin channel opening was reported to occur during hypoxia in endothelial cells with two thirds of ATP release connexin hemichannel mediated, but upon reperfusion, only Cx43 hemichannel opening occurred (Kim and Green, 2016). These studies indicate that Cx43 hemichannels likely do open after ischemia and may have an ongoing role in the evolution of injury after the insult. This is supported by the demonstration that prolonged infusion of Peptide5 (i.c.v., starting at 90 min) at a dose of 50 μmol/kg over 1 h, then 50 μmol/kg over 24 h of Peptide5 provided greater neuroprotection compared to a 1 h infusion only (50 μmol/kg) (Davidson et al., 2012). Furthermore, delayed administration of another known hemichannel blocker, Gap 26 (i.p., 50 μmol/kg) at 24 h after HI still had neuroprotective effects (Li et al., 2015). Interestingly, when Peptide5 administration (i.c.v., 50 μmol/kg for 1 h followed by 50 μmol/kg for 24 h) was delayed until 3 h after the end of ischemia in near-term fetal sheep, the neuroprotective effects were reduced when compared to earlier administration (Davidson et al., 2012, 2015a). The delayed administration reduced the seizure burden, but there was no clear effect on cell survival or EEG recovery (Davidson et al., 2012, 2015a).

The mechanisms behind how Cx43 hemichannels lead to neuronal and oligodendrocyte loss in HI is not well-understood. During and/or after HI there is impaired intracellular Ca^2+^ handling. This has been shown to contribute to mitochondrial dysfunction and necrotic and apoptotic cell death (Puka-Sundvall et al., 2000; Mallard et al., 2014). Potentially, the opening of hemichannels could lead to an influx of excessive intracellular Ca^2+^ accumulation leading directly to the neuronal and/or oligodendrocyte death (Galinsky et al., 2018a). In support, GABAergic striatal neurons expressing intracellular calcium binding proteins are shown to be highly susceptible to HI injury in near-term fetal sheep (Galinsky et al., 2017).

ATP has been widely associated with both inflammasome signal 1 priming which results in transcriptional upregulation of pro-IL-1β and pro-IL-18 and molecules in the inflammasome pathway itself, and inflammasome signal 2 activation that results in assembly of the NLRP3-ASC-pro-caspase1 inflammasome complex within the cell cytoplasm, and activation of caspase 1. Cx43 hemichannel mediated ATP release in particular has been associated with NOD-like receptor protein-3 (NLRP3) inflammasome complex assembly as shown in retinal pigment epithelial cells (Mugisho et al., 2018), and with inflammasome activation *in vivo* in a model of chronic pain (Tonkin et al., 2018). Neuron and oligodendrocyte damage after perinatal brain injury may also be due to this innate immune system inflammatory response, and is discussed further in section Activation of the Inflammasome below.

## Pannexin Channels

Pannexin channels may have similar functions to connexin hemichannels, but their role in perinatal brain injury is not well-understood. Pannexins share homology with the gap junction proteins in invertebrates called innexins (Panchin, 2005), and have a similar topological structure to connexins, despite not sharing sequence homology (Panchin, 2005) (Figure 1). Unlike connexins, pannexins have N-glycosylation on the extracellular loop, which appears to prevent the formation of cell-cell junctions (Boassa et al., 2007) although there is some evidence for pannexin junction formation in C2C12 cells in culture (Ishikawa et al., 2011). There are three subtypes of pannexin, of which pannexin 1 (Px1) and pannexin 2 (Px2) are found to be expressed in the adult and developing brain (Bruzzone et al., 2003; Vogt et al., 2005). Px1 is predominantly expressed in the plasma membrane, whereas Px2 is mainly expressed in intracellular membranes (Boassa et al., 2007).

Opening of pannexin channels can be induced by a range of stimuli *in vitro*—including OGD, high extracellular K^+^ concentration, hypoglycemia, cell swelling and the stimulation of NMDA receptors (Thompson et al., 2006, 2008; Kawamura et al., 2010; Santiago et al., 2011). Px1 channels are permeable to ions, and molecules, such as ATP and glucose (Bruzzone et al., 2003; Bao et al., 2004; Riquelme et al., 2013) and the opening of Px1 channels contributes to anoxic depolarization, which can lead to cell death (Thompson et al., 2008; Weilinger et al., 2012). In astrocytes on the border of abscess regions caused by S. aureus intracerebral inoculation in mice, there is increased Cx43 hemichannel and Px1 channel activity (Karpuk et al., 2011). Increased Px1 channel activity in apoptotic lymphocytes *in vitro* has been associated with providing “find me” signals through ATP release to attract macrophages (Chekeni et al., 2010). In addition, Px1 channel opening has been linked to the activation of the NLRP3 inflammasome and IL-1β release in astrocytes subjected to OGD (Jian et al., 2016).

In adult rodent models of stroke, blocking Px1 has neuroprotective effects (Cisneros-Mejorado et al., 2015a; Wei et al., 2015; Freitas-Andrade et al., 2017). However, to the best of our knowledge, the effect of Px1 blockade after HI has not been examined in the developing brain. Mefloquine (Px1 blocker) injections given during the start of middle cerebral artery occlusion (MCAO) (i.p., 1 mg/kg/day) in adult mice reduced infarction size and improved motor scores (Cisneros-Mejorado et al., 2015a). It should be noted, however, that mefloquine may also inhibit connexin hemichannel opening (Cruikshank et al., 2004). The effectiveness of Px1 blockade is time dependent, as the administration of probenecid, another non-specific Px1 blocker, was most protective against the death of hippocampal CA1 neurons when given before reperfusion (i.v., 2 mg/kg) in adult rats subjected to MCAO (Wei et al., 2015). There was partial protection when administered at 2 h, but no effect when delayed until 6 h after the insult (Wei et al., 2015). Probenecid administration for 7 days starting at 6 h, however, was more protective than a single dose at 6 h. There was also reduced inflammation, shown by reduced astrocytic and microglial immunoreactivity (Wei et al., 2015). The role of Px1 may also be sex dependent, as Px1 protein levels were higher in female than male mice before and after MCAO (Freitas-Andrade et al., 2017). Both Px1 knockout mice and probenecid treated mice (at 1.5 and 5 h, 250 mg/kg, i.p.), had reduced infarct volumes after MCAO compared to control, but the effect was not seen in males (Freitas-Andrade et al., 2017). It is unclear what mechanisms are involved in producing this sex difference, but it may be related to differences in caspase-dependent cell death pathways and estrogen receptor β signaling in females (Freitas-Andrade et al., 2017).

The interaction of connexin hemichannels and pannexin cell channels has been examined in astrocyte cultures exposed to hypoxia and artificial cerebral spinal fluid medium that mimics ischemic conditions in the brain (Orellana et al., 2010). After reoxygenation, there was increased hemichannel activity, indicated by increased uptake of ethidium bromide, which peaked at 1 h after rexoygenation. This hemichannel activity was mediated by Cx43 hemichannels and not pannexin channels, as dye uptake was reduced by Cx43 hemichannel blockade or Cx43 knockout, but not pannexin blockade (Orellana et al., 2010). This is consistent with the endothelial cell hypoxia-reperfusion study referred to above, where pannexin channel opening was reported during hypoxia, but not reperfusion (Kim and Green, 2016). Furthermore, connexin hemichannels, but not pannexin channels, have been implicated in the inflammasome pathway in muscular dystrophy (Cea et al., 2016), and inflammatory cytokine release normally associated with the inflammasome pathway in rodent models of Parkinson's disease (Maatouk et al., 2018) and Alzheimer's disease (Yi et al., 2016). Finally, pannexin channels are self-regulated, and whilst they may release ATP, they are also closed by the presence of ATP in the extracellular milieu (Qiu and Dahl, 2009). Taken overall, it is possible that pannexin channel opening may play a role in inflammasome pathway initiation, but amplification and perpetuation in chronic disease conditions may be primarily connexin hemichannel mediated. This is likely to apply to perinatal brain injury and inflammation too, but remains to be proven.

## Astrocytes and Inflammation

Astrocytes play an important physiological role in maintaining the homeostasis of the microenvironment in the brain. Astrocytes are the most abundant cell type and account for half of the cells in the central nervous system (Markiewicz and Lukomska, 2006). They are electrically non-excitable cells, but have a role in propagating Ca^2+^ waves through gap junctions between astrocytes-astrocytes and astrocytes-neurons (Nedergaard, 1994), and extracellularly between isolated cells (Hassinger et al., 1996). Astrocytes maintain K^+^ ionic balance (Wallraff et al., 2006), and have an important role in the reuptake and recycling of glutamate (Rothstein et al., 1994). Additionally, astrocytes provide metabolic support for neurons by producing lactate, which is taken up by neurons to produce ATP (Mächler et al., 2016).

Astrocytes can also contribute to the inflammatory response after perinatal brain injury. Reactive astrogliosis can occur where astrocytes undergo hypertrophy, increase expression of glial fibrillary protein and form a glial scar around a focal injury (Romero et al., 2014). However, astrogliosis responses have varied between different experimental paradigms. GFAP expression was increased but numbers of GFAP positive cells did not change in P7 rats at 24 h after HI (Odorcyk et al., 2017). There were increased GFAP positive cells and area fraction in preterm fetal sheep at 3 days after umbilical cord occlusion (Wassink et al., 2017). In contrast, there were no changes in the number and area fraction of GFAP positive cells in near-term fetal sheep at 7 days after carotid artery occlusion (Davidson et al., 2016). Moreover, there was a reduction in area fraction and the size of astrocytes in neonatal pigs at 3 days after hypoxia (Sullivan et al., 2010).

Astrocytes predominantly express Cx43, with some expression of Cx26, Cx30, Cx40, and Cx45 (Nagy et al., 1997, 2003; Dermietzel et al., 2000). The opening of astrocytic Cx43 hemichannels after HI may compromise the ability of astrocytes to maintain homeostasis and neuronal support. This in turn, can have detrimental effects on neuronal and oligodendrocyte survival, the propagation of seizures and secondary energy failure (Davidson et al., 2013a). Additionally, astrocytes cultures subjected to OGD/reperfusion increased ATP release through Cx43 hemichannels. Further, treating OGD/reperfusion microglial cultures with ATP or medium from astrocytes subjected to OGD/reperfusion induced microglial activation (Yin et al., 2018b). The potential mechanisms of microglial activation through ATP signaling is discussed further below.

## Microglia and Inflammation

Microglia are the innate immune cells of the brain; having an important role in immune surveillance under normal conditions (Nimmerjahn et al., 2005). Aside from their immune function, they are important for apoptosis and pruning of excessive neurons and synapses, and the phagocytosis of debris during brain development (Baburamani et al., 2014). After brain injury, however, microglia undergo proliferation and activation during the inflammatory response (Baburamani et al., 2014). Resting microglia commonly have ramified morphology and become amoeboid when activated. However, during development, microglia migrate in the brain in amoeboid form and change into a ramified morphology (Pierre et al., 2017).

Activated microglia have various roles, where the classically activated, or M1-like polarization facilitates the progression of inflammation and the alternatively activated, or M2-like polarization is involved in the resolution of inflammation (Bonestroo et al., 2013; Jaworska et al., 2017). The M1-like microglia express cluster of differentiation (CD)86, CD16, CD32, produce reactive oxygen species and nitric oxide synthase, proteases, a range of interleukins and inflammatory cytokines, such as IL-1β, IL-6, and TNF-α (Czeh et al., 2011; Barakat and Redzic, 2015; Fumagalli et al., 2015). The M2-like polarization express CD206 and arginase 1 and produce anti-inflammatory IL-10 and growth factors, favoring repair (Czeh et al., 2011; Barakat and Redzic, 2015; Fumagalli et al., 2015). However, the view that there is a clear polarization of the microglia phenotypes has been challenged in recent years, and currently it is hypothesized that microglial polarization is a continuum with M1 on one end, and M2 on the other (Cherry et al., 2014; Hellström et al., 2016).

In P7 rats, there was a marked increase in CD45/CD11b positive cells in injured areas at 24 h after transient MCAO. These cells were predominantly microglia and not infiltrated blood monocytes (Denker et al., 2007). Supporting this, microglia/macrophage counts were significantly elevated in the hippocampus from 1 day after common carotid artery ligation and hypoxia in P9 mice (human term brain maturation equivalent) (Ferrazzano et al., 2013). There may be a biphasic response of microglial upregulation, with an initial increase in microglial number in the hippocampus at 2 days, followed by a delayed increased in the striatum and cortex at 9 days, after common carotid artery ligation and hypoxia in P9 mice (Cikla et al., 2016). Similarly, classically activated CD11b/CD86 positive microglia from whole brain homogenates were upregulated at 24 h, followed by a secondary peak at 1 week, which was resolved to control levels by 2 weeks after HI (Rice-Vannucci model) in P10 mice (Winerdal et al., 2012). However, the alternative microglial activation markers were not assessed in that study. Significantly elevated counts of ionized calcium-binding adapter molecule 1 (Iba1) positive microglia in the intragyral white matter of the first and second parasagittal gyrus, and the periventricular white matter were present after 1 week of recovery from global cerebral ischemia in near-term fetal sheep (Davidson et al., 2015c, 2016). Moreover, Iba1 positive microglial number was still elevated in the same white matter regions as above at 21 days after severe HI induced by acute umbilical cord occlusion in preterm fetal sheep (van den Heuij et al., 2019). This longer term recovery highlights the chronic nature of inflammation after perinatal HI insults.

The functions of activated microglia appear to change over time after HI. The activation characterization of microglia and infiltrated macrophages has been assessed in P9 mice after common carotid artery occlusion followed by hypoxia (Hellström et al., 2016). There was an increase in mRNA levels of both pro and anti-inflammatory genes at 24 h after the insult. Furthermore, there was a significant increase in classically activated CD86 positive microglia. In contrast, although absolute numbers of alternatively activated CD206 positive microglia increased, their relative proportion was reduced. Surprisingly, there was a population of microglia which did not express CD86 or CD206, highlighting the complexity of microglia activation polarization (Hellström et al., 2016). The early rise of pro-inflammatory markers is supported by an increase of IL-1β and TNF-α mRNA expression between 1 and 24 h after HI in P7 rats (Rice-Vannucci model) (Bona et al., 1999). In another study, using the same paradigm to induce HI in P7 rats, both pro-inflammatory (IL-1β, TNF-α) and anti-inflammatory (IL-10) mRNA expression increased at 3 h after the insult. At 24 h, TNF-α expression was still elevated, but was lower than at 3 h. In addition, anti-inflammatory cytokine TGF-β expression also increased at 24 h along with an increase of CD206 and Iba1 positive microglia (Bonestroo et al., 2013). However, in P7 rats, the majority of CD86 positive microglia were co-localized with IL-1β and only a small number were co-localized with arginase-1, at 6 days after carotid artery ligation and hypoxia (Jaworska et al., 2017). Although some studies indicate a trend toward resolution of inflammation over time, microglia remain upregulated even 3 weeks after HI in preterm fetal sheep (van den Heuij et al., 2019), and so further experimental studies are needed to determine the function of microglia at these later time points.

The increase in inflammatory cytokine release highlighted in the studies above is consistent with NLRP3 inflammasome activation (Shao et al., 2018). Both astrocytes and microglia are known to play key roles in the inflammasome pathway (for review see Song et al., 2017).

## ATP Release From Membrane Channels

**Figure 2:**
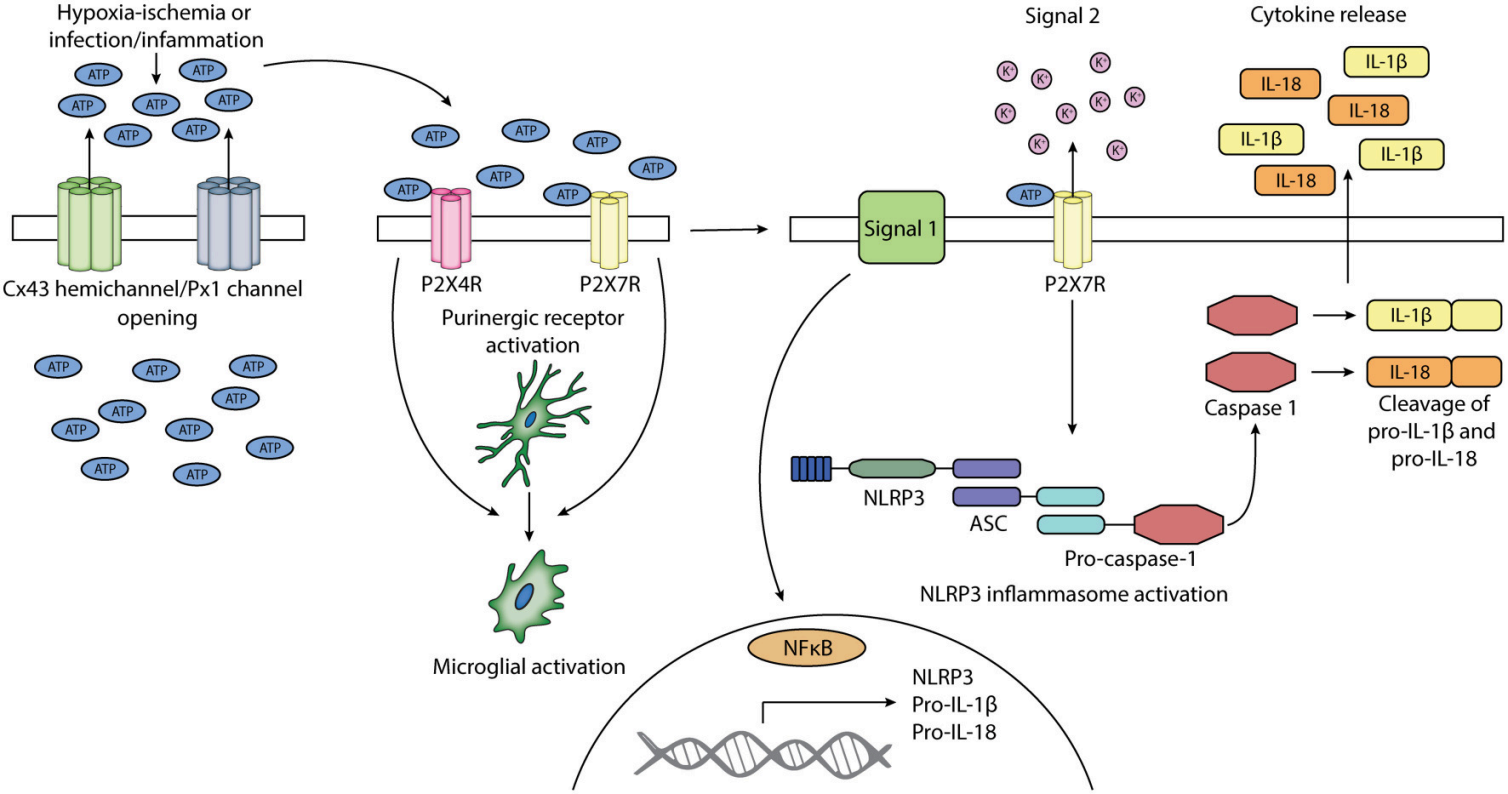
Schematic diagram of the potential involvement of connexin 43 hemichannels and pannexin 1 channels and purinergic receptors P2X4 and P2X7 in the activation of microglia and NLRP3 inflammasome. Cx43 hemichannels and Px1 channels may open after exposure to HI or infection/inflammation. The opening of these channels leads to the release of ATP into the extracellular space. Extracellular ATP can activate purinergic receptors P2X4R and P2X7R. The activation of P2X4R and P2X7R can lead to the activation of microglia. Signal 1 of NLRP3 inflammasome activation is provided by PAMPs or DAMPs, which leads to the upregulation of NLRP3, pro-IL-1β, and pro-IL-18. Signal 2 is mediated by ATP activation of P2X7R and can lead to the assembly of NLRP3, ASC, and pro-caspase 1, forming the NLRP3 inflammasome. Pro-caspase 1 is activated into caspase 1, which cleaves pro-IL-1β and pro-IL-18 into their mature forms. Mature IL-1β and IL-18 is secreted.

Both connexin hemichannels and pannexin channels can contribute to ATP release into the extracellular space (Kang et al., 2008; Chekeni et al., 2010; Orellana et al., 2011; Bennett et al., 2012), although, as discussed above, pannexin channels may also be regulated (closed) by extracellular ATP (Qiu and Dahl, 2009). ATP is a high energy molecule, essential for driving many cellular processes in the body. It also has a crucial neurotransmitter and neuromodulatory role in the brain (Melani et al., 2005; Pedata et al., 2016). ATP can be co-released with other neurotransmitters or function as an extracellular signaling molecule on its own (Burnstock, 2009; Suurväli et al., 2017). However, under pathological conditions, it may have injurious effects acting as a DAMP (Melani et al., 2005; Pedata et al., 2016) but more crucially as an inflammasome signal 2 activator. Extracellular ATP can activate purinergic receptors, which can perpetuate inflammasome activity in a number of brain cell types, including microglia (Bours et al., 2011) (Figure 2).

The bulk of ATP production occurs in mitochondria through oxidative metabolism, but a small amount is produced through anaerobic glycolysis. The disruption of oxygen and glucose delivery during HI affects oxidative metabolism, attenuating the production of ATP (Bainbridge et al., 2014; Wassink et al., 2014). ATP is critical for the function of Na^+^/K^+^-ATPase, which maintain ionic gradients. When ATP is depleted, the failure to maintain cellular ionic homeostasis causes cytotoxic edema, which is indicated by a rise in cortical impedance starting soon after the onset of ischemia in near-term fetal sheep (Williams et al., 1993; Davidson et al., 2018b). We have previously shown that during profound asphyxia in near-term fetal sheep, there was a rapid increase in oxidized cytochrome oxidase at 2–6 min from the start of asphyxia (Drury et al., 2012). The increase in oxidized cytochrome oxidase indicate that there was a decreased availability of reducing equivalents in the mitochondrial electron transport chain, impairing ATP production. Additionally, a phosphorus magnetic resonance spectroscopy study showed that cerebral phosphocreatine and total nucleotide triphosphate decreased, whereas inorganic phosphate increased during HI in neonatal piglets (Bainbridge et al., 2014). During secondary energy failure at 24 and 48 h after HI in neonatal piglets, the cerebral phosphocreatine/inorganic phosphate and nucleotide triphosphate/exchangeable phosphate pool also decreased (Lorek et al., 1994).

Paradoxically, at a time when ATP production is impaired, there is augmented ATP release into the extracellular space after permanent MCAO in the adult rat brain (Melani et al., 2005, 2012). Microdialysis samples collected from the striatum showed that ATP concentrations were an average of 30 nmol/L before ischemia, which increased to an average of 50 nmol/L after permanent MCAO (Melani et al., 2012). When an ecto-ATPase inhibitor—hexapotassium dihydrogen monotitanoundecatungstocobaltate (II) tridecahydrate (PV4, 100 μmol/L) was used, the extracellular ATP concentrations were between 320 and 450 nmol/L (Melani et al., 2012). This is within the range of what was detected in hippocampal slices during reoxygenation after OGD (700 nmol/L), using a biosensor measured in real time (Frenguelli et al., 2007). However, extracellular ATP release has not been examined after HI in the developing brain.

The increase in extracellular ATP can lead to a subsequent increase in adenosine, as ATP is catabolized into adenosine, through the ATP → ADP → AMP → adenosine pathway, mediated by ecto-nucleotidase enzymes (Pedata et al., 2016). Adenosine concentrations increased by 90% during a 1 h period of fetal hypoxia induced by reduction of maternal fraction of inspired oxygen, compared to levels during normoxia in 0.8 gestation fetal sheep (Koos et al., 1997). The rise in adenosine concentrations was attributed to the degradation of AMP, as blocking the degradation of AMP to adenosine with adenosine 5′-(α,β-methylene) diphosphate (AOPCP), an ecto-5′-nucleotidase inhibitor, attenuated the hypoxia-induced rise in adenosine (Koos et al., 1997). In support, in the first hour after permanent MCAO in the adult rat brain, extracellular adenosine was mainly derived from ATP hydrolysis (Melani et al., 2012). This is in contrast to physiological conditions, when adenosine can be directly released from intracellular stores (Melani et al., 2012). Adenosine is very important to the initial metabolic suppression that occurs at the beginning of an HI insult (Hunter et al., 2003). Blocking this response with the adenosine A1 receptor antagonist 8-cyclopentyl-1,3-dipropylxanthine (DPCPX) (2.5 mg/mL) in near-term fetal sheep before and during profound asphyxia, was associated with increased neuronal loss compared to the vehicle group (Hunter et al., 2003). Further studies are needed to examine the interaction of ATP and adenosine *in vivo*, in the developing brain after HI, and to investigate inflammasome complex assembly under those conditions. In parallel, it is necessary to consider the purinergic receptors themselves (see section Purinergic Receptors).

## Purinergic Receptors

Purinergic P1 and P2 receptors are activated by adenosine and ATP, respectively. Within the group of P2 receptors, there are P2X receptors, which are ionotropic and P2Y, which are metabotropic (Pedata et al., 2016). In this review, we will focus on the P2X receptors, which form trimeric cationic channels in the cell membrane (Habermacher et al., 2016). Of the seven P2X subtypes (P2X1-7), P2X4 receptor (P2X4R), and P2X7 receptor (P2X7R) have been more widely studied in the brain (Pedata et al., 2016). These channels open in response to ATP binding and are permeable to Na^+^, K^+^, and especially Ca^2+^ (Hattori and Gouaux, 2012). The activation of purinergic receptors can lead to the opening of more connexin hemichannels (and at least potentially pannexin channels), triggering further release of ATP in a process known as “ATP-induced ATP release” in a positive feedback loop (Stout et al., 2002; Baroja-Mazo et al., 2013).

### P2X4 Receptor

P2X4R is one of the most sensitive P2X receptors, as it binds ATP in the nanomolar range, whereas P2X7R binds at a micromolar range (Suurväli et al., 2017). It also has the highest permeability to Ca^2+^, and is the most abundantly expressed P2X subtype in the brain (Egan and Khakh, 2004; Cheng et al., 2014), found on neurons and glial cells, in various brain regions (Bo et al., 2003; Stokes et al., 2017; Suurväli et al., 2017). Under physiological conditions, P2X4R may have an important role in neurotransmission pathways (Suurväli et al., 2017). P2X4 knockout mice show deficits in sensorimotor tasks and social interactions, which was associated with altered subunit expression of glutamate and gamma-aminobutyric acid (GABA) receptors (Wyatt et al., 2013). As P2X4R is expressed abundantly in cells of myeloid origin, such as microglia and monocytes, they may play important roles in pro-inflammatory cytokine release following injury (Cavaliere et al., 2003).

P2X4R has been targeted for neuroprotection after HI. Global P2X4R knockout mice have a reduction in infarct volume and IL-1β and TNF-α release after MCAO compared to wild type mice (Verma et al., 2017). However, when P2X4R was knocked out in myeloid cells only, the neuroprotective effect was only seen in females. The role of P2X4R is complex, as the P2X4R knockout was associated with increased depressive-like behavior compared to wild type mice measured at 30 days after MCAO (Verma et al., 2017). In addition, P2X4R on vascular endothelial cells was involved in neuroprotection via ischemic preconditioning after MCAO in adult mice, as the inhibition of P2X4R abolished this effect (Ozaki et al., 2016).

### P2X4 Receptor and Activation of Microglia

Microglia and peripheral monocytes have been shown to express P2X4R, and electrical currents can be induced by ATP activation of P2X4R (Wang et al., 2004; Cheng et al., 2014). The expression of P2X4R is upregulated in organotypic hippocampal culture at 24 h after OGD (Cavaliere et al., 2003) In P0 rats, P2X4R protein expression increased from 4 h after hypoxia until 14 days (Li et al., 2011). However, in P3 rats, there was a significant increase in P2X4R protein expression only at 5 days after common carotid artery occlusion and hypoxia (Wixey et al., 2009). In both neonatal rat studies, P2X4R expression was predominantly colocalized with microglia (Wixey et al., 2009; Li et al., 2011). Although the increase in P2X4R at 5 days was not temporally correlated with the early increase in Iba1 protein expression seen at 2 days after HI, it correlated with the delayed increase in Iba1 protein expression from 6 days after HI. It was postulated that these P2X4R positive microglia may be a distinct population of microglia involved in neuroinflammation (Wixey et al., 2009). Furthermore, P2X4R blockade with trinitrophenyl (TNP)-ATP (20 μM) prevented the change of primary microglial cells to a more rounded morphology after hypoxia, and attenuated the increased IL-1β and TNF-α protein expression (Li et al., 2011).

### P2X7 Receptor

Although, P2X7R has a lower affinity of 0.1–1 mM for ATP, when compared to P2X4R, the increased extracellular ATP levels after ischemia may be sufficient to activate it (Surprenant and North, 2009; Melani et al., 2012). The deleterious roles of the P2X7R include increasing intracellular Ca^2+^ concentrations, glutamate release and inflammasome activation (Di Virgilio, 2007; Matute et al., 2007; Rossi and Volterra, 2009; Ye et al., 2017). Additionally, prolonged ATP activation of P2X7R can transform the cationic channel into a large membrane pore, permeable to molecules 900 Da in size (Yan et al., 2010).

Animal studies have shown that P2X7R is upregulated after ischemia in the immature and adult brain. In a fetal rat model of intrauterine asphyxia, P2X7R expression in the hippocampus was upregulated 3-fold immediately after asphyxia, but returned to control levels at 60 min (Frizzo et al., 2010). In adult rats, however, cortical P2X7R expression was increased 5-fold compared to controls at 4 days after permanent MCAO (Franke et al., 2004). Whereas, in adult mice subjected to photothrombotic cerebral ischemia, cortical P2X7R expression was upregulated from 1 day post-ischemia until 5 days (Ye et al., 2017). In cortical neuronal culture, there was a significant upregulation of P2X7R after exposure to OGD (Ye et al., 2017). P2X7R expression was increased in the CA1 region of the hippocampus from 6 h and maintained until 7 days after global cerebral ischemia in adult rats (Yu et al., 2013). The differences in the timing of P2X7R upregulation could be due age, species, brain region and type of insult.

Blocking P2X7R has been shown to be beneficial for ischemic brain injury *in vitro* and in adult animals. In neuronal culture, Brilliant Blue G—a P2X7R blocker (50 nM or 5 μM), applied during OGD, reduced cell death measured at 24 h after OGD (Arbeloa et al., 2012). This was replicated *in vivo*; Brilliant Blue G (30 mg/kg, i.p.) administered at 30 min during a 90 min MCAO and continued daily in adult rats reduced infarct volume and neuronal loss when measured at 3 days after the insult (Arbeloa et al., 2012). In support, Brilliant Blue G treatment (50 mg/kg, i.v.) administered daily, for 3 days, starting immediately after occluding bilateral common carotid and vertebral arteries in adult rats, partially attenuated CA1 neuronal loss in the hippocampus at 7 days after injury (Yu et al., 2013). Although Brilliant Blue G has higher affinity for P2X7R, it can also bind to other P2X receptors at higher concentrations (Chu et al., 2012). However, the administration of a more selective P2X7R antagonist—A-438079 (3 μg, i.c.v.), prior to transient common carotid artery occlusion in adult rats, had similar neuroprotective effects to Brilliant Blue G (10 μg, i.c.v.) and Oxidized ATP (1 μg, i.c.v.), in terms of neuronal survival and motor performance (Chu et al., 2012). Additionally, in an adult rat model of intracerebral hemorrhage, silencing the P2X7R gene reduced NLRP3 inflammasome activation and release of IL-1β and IL-18 at 24 h after the insult (Feng et al., 2015). It is difficult to disentangle the timing of when P2X7R is involved in injury, as most of these studies above started blocking P2X7R either during or immediately after ischemia.

Several studies have shown that connexin hemichannels and pannexin channels are involved in purinergic signaling. For example, IL-1β release induced by P2X7R activation was associated with Px1 channels in macrophages (Pelegrin and Surprenant, 2006). When a Px1 blocker—mefloquine (1 mg/kg, i.p.) was co-administered with Brilliant Blue G (30 mg/kg twice per day, i.p.) to block P2X7R, starting at 30 min during 60 min of MCAO, there was no additive benefit when compared to administering either drug alone in adult mice after MCAO (Cisneros-Mejorado et al., 2015a). However, as stated earlier, mefloquine can also target connexin hemichannels (Cruikshank et al., 2004). A potential mechanism of injury may be ATP release, as both the prevention of ATP release, or inhibition of P2X7R may have beneficial effects (Cisneros-Mejorado et al., 2015b). For example, targeting Cx43 using Peptide5 at 0 h (10 mg/kg), 2 h (5 mg/kg), and 4 h (2.5 mg/kg, i.p.), after spinal cord injury in rats was associated with a reduction is tissue damage and improved functional recovery (Mao et al., 2017). Similarly, the administration of Brilliant Blue G to inhibit P2X7R (10 or 50 mg/kg daily for 3 days) starting 10–15 min after spinal cord injury in rats, reduced tissue damage and improved motor performance (Peng et al., 2009).

### P2X7 Receptor and Activation of Microglia

In adult rats after MCAO, the expression of P2X7R was colocalized with microglia at 1 and 4 days, neurons at 4 and 7 days, and astrocytes at 4 days after the insult (Franke et al., 2004). *De novo* expression of P2X7R was observed on activated microglia at 24 h after MCAO in adult rats, with no expression seen in control animals (Melani et al., 2006). It has been debated as to whether the overexpression of P2X7R drives the activation of microglia, or whether the overexpression of P2X7R is a result of microglial activation (Bai and Li, 2013). In support of the role of P2X7R in microglia activation, the overexpression of P2X7R by transfection of microglia in culture resulted in activation and proliferation of microglia (Monif et al., 2009). Additionally, P2X7R knockout mice showed significantly attenuated microglial activation at 72 h following MCAO compared to wild type mice (Kaiser et al., 2016). In the developing brain, both P2X7R and P2X4R were constitutively expressed in microglia in P3 rats, but P2X4R immunofluorescence was more intense than P2X7R. However, only the changes in P2X4R expression was examined after exposure to hypoxia in P0 rats (Li et al., 2011).

## Activation of the Inflammasome

The family of PRRs include Toll-like receptors (TRLs), NOD-like receptors (NLRs), and retinoic acid-inducible gene-I-like receptors (RLRs) which recognize PAMPs or DAMPs (Hagberg et al., 2015; Mallard et al., 2018). There is growing interest in the role of NLRs in inflammatory diseases, but relatively little is known about their role in perinatal brain injury. Inflammasomes are a multimeric complex of proteins which oligomerize after sensing PAMP or DAMP signals (Jo et al., 2016). Typically, NLR inflammsomes are formed by the assembly of the NLR family protein, apoptosis-associated speck-like protein containing a caspase recruitment domain (ASC) and pro-caspase 1. The 22 members of the NLR family are distinguished by their N-terminal effector domains, and grouped into NLRA, NLRB, NLRC and NLRP (Ting et al., 2008). One of the better characterized inflammasomes in the NLR family is the NOD-like receptor protein-3 (NLRP3) inflammasome. The activation of the NLRP3 inflammasome leads to the activation of caspase 1, the cleavage and release of mature IL-1β and IL-18 and subsequent release of other cytokines (Jo et al., 2016; Mugisho et al., 2018).

There are two steps to NLRP3 inflammasome activation. Signal 1 is a priming step, induced by PAMPS, such as microbial toxins and surface proteins, TLR ligands and viral RNA (Martinon et al., 2009; Jun et al., 2012; Chakrabarti et al., 2015) or DAMPs, such as amyloid-β, hyaluronan, monosodium urate, calcium pyrophosphate dehydrate and extracellular ATP (Martinon et al., 2009; Gong et al., 2018). Endogenous inflammatory cytokines including TNF-α and IL-1β can also act as priming signals (Franchi et al., 2009; He et al., 2016; Gong et al., 2018). The priming signal leads to the upregulation of NLRP3 and pro-IL-1β and pro-IL-18 expression through the nuclear factor kappa-light-chain-(NF-κB) translocation to the nucleus to trigger their transcription (He et al., 2016). Signal 2 of the NLRP3 inflammasome is the activation signal. This leads to assembly of the inflammasome complex. A clear model for activation remains unclear; much of the literature suggests that many of the same molecules involved in priming may have a secondary role in activation (Shao et al., 2018). That seems unlikely. Thus, is remains a significant question to define which molecular mechanism(s) trigger activation of the NLRP3 inflammasome (He et al., 2016; Jo et al., 2016). More recently, the list of potential activator signals has been narrowed down. Key players are now suggested to include: ATP, calcium signaling, and reactive oxygen species (He et al., 2016; Jo et al., 2016; Groslambert and Py, 2018). Mitochondrial dysfunction, lysosomal rupture and K^+^ efflux are also reported to be activators (He et al., 2016; Gao et al., 2017), although it is unclear to what extent some of these may be effects of inflammation rather than initiators *per se*.

Potassium efflux can be mediated through the opening of endogenous ion channels or through bacterial pore forming toxins (Gao et al., 2017). Purinergic receptors have been associated with the efflux of K^+^ (Yan et al., 2008) as have connexin hemichannels (Schalper et al., 2010; Leybaert et al., 2017). It is unclear how the reduction in intracellular K^+^ leads to the activation of the inflammasome although studies have shown that it is a necessary step for the kinase Never In Mitosis A-Related Kinase 7 to bind to NLRP3, required for the assembly of the inflammasome (He et al., 2016; Shi et al., 2016). Additionally, K^+^ efflux can lead to the disruption of mitochondrial function and production of mitochondrial reactive oxygen species (Tang et al., 2017). It is also of note that potassium efflux is induced by ATP suggesting that ATP release may therefore be the primary or upstream activator not only for potassium efflux, but also mitochondrial dysfunction and reactive oxygen species. Our work has shown that reducing ATP release on its own alone (by blocking connexin hemichannels alone) is sufficient to shut down inflammasome complex assembly, while the addition of exogenous ATP sufficient to reestablish assembly (Mugisho et al., 2018), consistent with the evidence that purinergic receptors play a key role, as outlined above and in Table 1.

### Inflammasome Activation in Brain Injury

A large body of evidence in the adult brain shows that inflammation is a least in part mediated by the NLRP3 inflammasome in animal models of stroke, traumatic brain injury and subarachnoid hemorrhage with upregulation of mRNA or protein expression of NLRP3, ASC, caspase-1 and associated inflammatory cytokines, such as TNF-α, IL-18 and IL-6 (Gao et al., 2017; Ye et al., 2017; Ismael et al., 2018; Lee et al., 2018; Wang et al., 2018; Xu et al., 2018; Yin et al., 2018a). Additionally, clinical data show that children with severe traumatic brain injury (open/closed head injury not specified) had elevated levels of NLRP3 in cerebrospinal fluid collected between 0 to ~72 h after the injury (Wallisch et al., 2017). NLRP3 and IL-1β mRNA expression was increased in the hippocampus, striatum and the thalamus at 24 h after common carotid artery ligation followed by hypoxia in P9 neonatal mice (Ystgaard et al., 2015). Supporting this, NLRP3 immunofluorescence increased at 24 h after common carotid artery ligation and hypoxia in P7 rats, which was predominantly colocalized with microglia (Chen et al., 2018). Additionally, there was an increase in IL-1β, caspase 1 and NLRP3 protein expression (Chen et al., 2018).

Targeting the NLRP3 inflammasome has neuroprotective effects in ischemic brain injury either through selective inhibition of NLRP3, or through anti-inflammatory drugs which affect the NLRP3 pathway (Ye et al., 2017; Chen et al., 2018; Ismael et al., 2018). The selective inhibition of NLRP3 using MCC950 (50 mg/kg, i.p. *in vivo* and 1 μM *in vitro*) reduced neuronal apoptosis when delivered after photothrombotic cerebral ischemia in adult mice, and before OGD in neuronal cultures (Ye et al., 2017). In support, MCC950 (50 mg/kg, i.p.) administration at 1 and 3 h was associated with a reduction in infarction size and cerebral edema, and improved neurological deficit scores, after MCAO in adult mice. These findings were in turn associated with a decrease in protein levels of NLRP3, caspase 1 and ASC at 24 h after the insult (Ismael et al., 2018). Ginkgolide B—a component of Ginkgo biloba extracts (5–10 mg/kg, i.p.), which has anti-inflammatory effects, was administered to P7 rats 30 min prior to common carotid artery ligation and hypoxia and was associated with the attenuation of the increased expression of NLRP3, caspase 1 and IL-1β at 24 h after injury. These changes were accompanied by reduced infarct size and cerebral edema measured at 72 h (Chen et al., 2018). In contrast, NLRP3 knockout was not associated with neuroprotection in P9 mice, as infarction volume was not significantly different compared to wild type mice at 24 h after common carotid artery ligation and hypoxia (Ystgaard et al., 2015) and in some cases other NLRs or other pathways may be implicated.

### Inflammasome Activation in Perinatal Infection/Inflammation

The studies discussed in this review predominantly focused on the role of connexin hemichannels and pannexin channels and purinergic receptors in experimental models of HI. Given that HI and infection/inflammation both lead to neuroinflammation, it is possible that both may be perpetuated through inflammasome pathway activation. In this section, we discuss the evidence for inflammasome activation in infection/inflammation in clinical and experimental studies.

Inflammasome activation, at least in adults, is an important inflammatory response to combat infection. For example, adult mice deficient in NLRP3, ASC, or caspase 1 have reduced survival rates compared to wild type mice after Group B Streptococcus infection (Costa et al., 2012). Group B Streptococcus is a Gram-positive bacterium that is common cause of life-threatening sepsis and meningitis in neonates and pregnant women (Henneke and Berner, 2006). Preterm and very-low-birthweight neonates are susceptible to neonatal sepsis likely due to increased risk of exposure to microbes (for example via mechanical ventilation, intravenous catheters and parenteral feeding). Secondly, they may have deficits in their innate and adaptive immune responses leading to insufficient inflammatory mediators (Wynn and Wong, 2010; Strunk et al., 2011). In view of the latter, a study of 21 preterm infants born 24–32 weeks showed that preterm umbilical cord blood monocytes, had a reduction in IL-1β secretion and an induction of NLRP3 expression, compared to term infants and adult peripheral monocytes after ATP/LPS stimulation (Sharma et al., 2015). However, IL-1β secretion from preterm peripheral blood monocytes (24–29 weeks of gestation) collected at an average of 15 post-natal days was comparable to adult monocytes, suggesting that the impairment in IL-1β secretion is restored shortly after birth (Sharma et al., 2015). A study of 72 neonates showed that IL-1β secretion from peripheral blood mononuclear cells did not differ between extremely preterm (born <28 weeks) and very preterm infants (28–32 weeks) on the fifth day of life. However, the peripheral blood mononuclear cells (collected within 24 h of late onset sepsis) from the extremely preterm group secreted higher levels of IL-1β, than the very preterm neonates (Zasada et al., 2018). It is unclear whether this gestational age dependent response is related to the higher counts of circulating mononuclear cells found in the extremely preterm group.

Conversely, the excessive activation of the inflammasome and release of pro-inflammatory cytokines in neonatal sepsis, can lead to multi-organ failure and death (Gentile et al., 2015). Caspase-1/11 knockout mice (P5-7) exposed to cecal slurry to induce polymicrobial intra-abdominal neonatal sepsis had greater survival rates compared to wild type mice (Gentile et al., 2015). Additionally, there was reduced IL-1β and IL-18 release at 2, 6, 18, and 24 h after the induction of sepsis. Surprisingly, genetic ablation of ASC or NLRP3 was not associated with a protective effect (Gentile et al., 2015). The knockout of ASC or NLRP3 may be insufficient for the complete elimination of caspase 1 activity as it is not solely activated by the NLRP3 inflammasome (Gentile et al., 2015). Further adverse effects are evidenced from infants who have rare genetic mutations leading to over activation of the NLRP3 inflammasome which manifests as neonatal-onset multi-system inflammatory disease (Aróstegui et al., 2010).

Recent evidence has demonstrated that increased inflammasome activity is associated with chorioamnionitis (Gomez-Lopez et al., 2017). A study of 70 pregnant women who had undergone preterm labor showed that the chorioamniotic membranes from those with acute histological chorioamnionitis compared to those without, had greater mRNA levels of inflammasome components, including NLRP3, and other NLR proteins, caspase 1, IL-1β, IL-18, and increased ASC and caspase 1 complex formation (Gomez-Lopez et al., 2017). Additionally, a study of amniotic fluid from 143 women showed that those who had undergone spontaneous preterm labor, with intra-amniotic infection/inflammation (positive culture for microorganisms in the amniotic fluid, or a white blood cell count of >100 cells/mm^3^) had significantly higher caspase 1 levels compared to those without intra-amniotic infection/inflammation, delivering at term or preterm (Gotsch et al., 2008). Furthermore, umbilical cord blood monocytes from preterm infants with histological chorioamnionitis showed reduced caspase 1 activity compared to those without histological chorioamnionitis (Sharma et al., 2015).

Although NLRP3 mediated inflammation may be implicated in many diseases, further study of its involvement in perinatal brain injury after HI and infection/inflammation is necessary. Disentangling the temporal effects of inflammation is particularly important, as discussed earlier the role of inflammation can be both beneficial and deleterious.

## Conclusion

Connexin and pannexin membrane channels can contribute to the evolution of perinatal brain injury. Connexin hemichannel, and to a lesser extent pannexin channel, blockade, has been associated with neuroprotection in a number of models of brain injury. A potential mechanism of injury perpetuated by connexin hemichannels could be release of what may be a key inflammasome signal 2 activator, ATP. Extracellular ATP activates purinergic receptors, which in turn have been shown to be involved with the activation of microglia and the inflammasome complex. Blockade of purinergic receptors P2X4 and P2X7 has been protective in adult animal models of stroke but further studies are required to investigate the involvement of purinergic receptors in the propagation of perinatal brain injury, and in particular the timing of their contribution to spreading injury. However, although P2X4R and P2X7R have been implicated in microglial activation following HI insults, and could be targets for modulating microglial responses after perinatal brain injury, connexin hemichannel perpetuation of the inflammasome would be upstream of both. Increasing evidence suggests that modulating the cascade upstream of inflammasome activation may attenuate brain injury mediated through inflammation. Inflammasome activation should be investigated further in perinatal brain injury, as it may be an important mediator of deleterious inflammatory responses.

## Author Contributions

JD and KZ conceptualized this topical review. KZ, JD, AG, LB, and CG undertook manuscript writing and preparation of figures. All authors reviewed and edited this manuscript.

### Conflict of Interest Statement

CG has intellectual property related to connexin hemichannel and pannexin channel modulation for the treatment of inflammatory disease and regulation of the inflammasome pathway and is a co-founder of OcuNexus Therapeutics, which has a focus on the treatment of chronic disease indications. The remaining authors declare that the research was conducted in the absence of any commercial or financial relationships that could be construed as a potential conflict of interest.
